# Construction of Novel *Saccharomyces cerevisiae* Strains for Bioethanol Active Dry Yeast (ADY) Production

**DOI:** 10.1371/journal.pone.0085022

**Published:** 2013-12-23

**Authors:** Daoqiong Zheng, Ke Zhang, Kehui Gao, Zewei Liu, Xing Zhang, Ou Li, Jianguo Sun, Xiaoyang Zhang, Fengguang Du, Peiyong Sun, Aimin Qu, Xuechang Wu

**Affiliations:** 1 Institute of Microbiology, College of Life Sciences, Zhejiang University, Hangzhou, Zhejiang Province, China; 2 State Key Laboratory of Motor Vehicle Biofuel Technology (Tianguan Group Co., Ltd.), Nanyang, Henan Province, China; University of Wisconsin - Madison, United States of America

## Abstract

The application of active dry yeast (ADY) in bioethanol production simplifies operation processes and reduces the risk of bacterial contamination. In the present study, we constructed a novel ADY strain with improved stress tolerance and ethanol fermentation performances under stressful conditions. The industrial *Saccharomyces cerevisiae* strain ZTW1 showed excellent properties and thus subjected to a modified whole-genome shuffling (WGS) process to improve its ethanol titer, proliferation capability, and multiple stress tolerance for ADY production. The best-performing mutant, Z3-86, was obtained after three rounds of WGS, producing 4.4% more ethanol and retaining 2.15-fold higher viability than ZTW1 after drying. Proteomics and physiological analyses indicated that the altered expression patterns of genes involved in protein metabolism, plasma membrane composition, trehalose metabolism, and oxidative responses contribute to the trait improvement of Z3-86. This work not only successfully developed a novel *S. cerevisiae* mutant for application in commercial bioethanol production, but also enriched the current understanding of how WGS improves the complex traits of microbes.

## Introduction

Bioethanol, a long considered suitable alternative to fossil fuel, has shown a sharp increase in production since 2006 [1,2]. *Saccharomyces cerevisiae* strains are the main microbes used for ethanol production because of their capability to efficiently convert hexose to ethanol and carbon dioxide [3-5]. Traditionally, yeast cells are cultured and propagated until the cell number desired for ethanol fermentation is achieved. This process begins with slant cultivation followed by gradual expansion of the cultivation scale in liquid medium to increase the volume. As an alternative to this hassle, many factories use commercial active dry yeast (ADY) to initiate ethanol fermentation. The application of ADY in fuel ethanol production cuts down initial lag time for fermentation and reduces yeast culture plant numbers and the risk of bacterial contamination [6].

To improve the economic and social benefits of bioethanol, novel technologies, such as very-high-gravity (VHG) and high-temperature ethanol fermentation, have been proposed [7,8]. While these approaches can reduce energy, condensate water consumption and increase fermentation efficiency, they also impose many stresses (e.g. osmotic pressure, high temperature, and high concentration of ethanol) on yeast cells. During the air-drying and rehydration of ADY, cells are exposed to fluctuating osmotic pressures and other stressors [9-12]. Thus, only *S. cerevisiae* strains that are able to tolerate multiple stresses can be made into commercial ADY for ethanol fermentation under stressful conditions.

Since the publication of the S. *cerevisiae* genome in 1996, many functional genomics studies have been carried out to investigate how this model unicellular organism responds and adapts to environmental stresses [13-16]. Tolerance to even a single stressor is usually controlled by gene networks that are difficult to modify through genetic engineering. To overcome this difficulty, global and comprehensive gene modification techniques have been demonstrated for improving the stress tolerance and fermentation properties of strains. Whole-genome shuffling (WGS) is such an approach in which gene recombination is achieved at different positions throughout the whole genome by recursive protoplast fusion or crossing [17]. In this process, genetic diversity is generated and resultant mutants with the desired phenotypes can be selected using specific screening methods [18,19]. Pinel et al. reported that genome shuffling through recursive population mating improved the tolerance of *S. cerevisiae* to spent sulfite liquor [20]. Shi et al. demonstrated that the thermotolerance of the species can be significantly enhanced using WGS [8]. Our previous studies have also suggested that WGS can improve the ethanol yield of *S. cerevisiae* under VHG conditions [21,22]. These reports suggest that WGS is a powerful tool for selection of the strain of interest, probably improved.

In the present study, an excellent industrial *S. cerevisiae* strain, ZTW1, was selected and subjected to a modified WGS process to construct a novel yeast strain that is applicable in commercial bioethanol ADY production. After three rounds of genome shuffling, we obtained mutants with improved tolerance to multiple stresses and ethanol titer compared with ZTW1. A comparative study of the mutant Z3-86 and its parent strain ZTW1 was also performed to elucidate the mechanisms underlying the improved traits of the mutant.

## Materials and Methods

### Strains and media

*S. cerevisiae* strains NY1300 (CICC 1300), NY1308 (CICC 1308), R12, A1-3 (Angel active dry yeast), ZK2 (CCTCC AY2012002), and ZTW1 (CCTCC M2013061) were used in this study. Yeast extract peptone dextrose (YPD) medium (10 g/L yeast extracts, 20 g/L peptone, and 20 g/L glucose, pH 5.5) was used for yeast cultivation. The fermentation medium was made from cassava flour using the double enzyme hydrolysis method. Briefly, a certain amount of cassava flour and water (ratio, 1:2) was mixed and α-amylase (8 U/g cassava) was added to the cassava flour mix. The cassava mash was kept in an autoclave at 95 °C for 4 h for full liquefaction. Then glucoamylase (200 U/g cassava flour) was used to saccharify the mash at 60 °C for 2 h. α-amylase (20,000 U/mL) and glucoamylase (100,000 U/mL) were donated by Novozymes,China. Urea (NH_2_CONH_2_; Sinopharm, Shanghai, China) of 0.5% concentration was added to the cassava mash as a nitrogen source for the yeast.

### Drying process

Yeast cells were cultured in 500 mL YPD at 30 °C at 200 rpm for 24 h with the initial OD (A600) of 0.1. Cells were centrifugally separated and four-fifth of the supernatant was removed. Sorbitan monoestearate and NaOH was added to the yeast cream as described by Garre et al.[10]. A Büchner funnel combined a vacuum pump was used to filtrate the residual supernatant of yeast cream to obtain a yeast cake. Then, the cake was extruded through a sieve (1mm diameter) to obtain yeast strands. The strands were dried in a thermostat drier at 42 °C to loss intracellular water. The dry weight of yeast cells were determined by drying to constant weight at 100 °C for 6 h.

### Modified genome shuffling process

Genome shuffling was conducted by treatment with methyl benzimidazole-2-yl-carbamate (MBC) and conduction of three rounds of sporulation and hybridization. MBC is a antimitotic drug and can cause random chromosomal aberration in *S. cerevisiae* cells [23] and thus used to produce mutants with genomic structural variations from ZTW1 in this study. Parent strain ZTW1 was grown in 25 mL YPD medium [initial OD (A600)=0.05] with 50 μg/mL MBC (Adamas-beta, Shanghai, China) to an OD (A600) of 2 and then collected by centrifugation. Cells (~3×10^8^) were then transferred into 25 mL sporulation medium (1% NaAc, 0.2% yeast extract, 0.1% glucose, and 0.2% KCl, at pH 6.0) for culture at 28 °C for 5 d, after which the spores (~5×10^8^) were purified and crossed randomly in 50 mL YPD medium for 36 h. The hybrids (~3×10^9^) were then centrifuged collected and dried until 92% of their water had been eliminated as described above. The dried cells were cultured in liquid YPD at 30 °C for 2 h for rehydration. The rehydrated cells were spread onto YPD plates containing multiple stressors (8% ethanol (v/v), 0.7 M NaCl, and cultured at 36°C) and the first 120 colonies to appear were randomly selected for ethanol fermentation test. The ten hybrids with the highest ethanol titers were selected for further genome shuffling. Procedures for the two succeeding rounds of sporulation and hybridization were similar to those adopted for the first round.

### Fermentation and metabolites

Yeast cells were pre-cultured in 50 mL YPD for 24 h at 30 °C and harvested. Cells were transferred to a cassava mash at a concentration of ~2×10^7^ cells/mL. Anaerobic fermentation was performed in 500 mL Erlenmeyer flasks under regular (cassava mash containing 224.5 g/L glucose at 33 °C for 60 h) or stressful (concentration of glucose was increased to 280 g/L and the fermentation temperature was 35 °C) conditions. The concentrations of ethanol, glucose, glycerol, and acetic acid were measured by a high-performance liquid chromatography (HPLC) instrument using an Aminex HPX-87H column (Bio-Rad Laboratories, Hercules, CA, USA) at 60 °C with 5 mmol H_2_SO_4_ as the mobile phase at a flow rate of 0.6 ml min^−1^. Peaks were detected by a refractive index detector (410 differential refractometer, Waters) and were identified and quantified by comparison to retention times of authentic standards.

### Determination of phenotypic stability of the selected mutant

The selected mutant was first grown in 25 mL YPD medium at 30 °C at 200 rpm with the initial cell density of 1.5×10^6^/mL for 18 h. Cells were transferred to a fresh 25 mL YPD (initial cell concentration was 1.5×10^6^/mL) and cultured at 30 °C at 200 rpm for 12. Such passage was repeated for sixty times. The cells from the twentieth, fortieth, and sixtieth generation were diluted and spread onto YPD plates. Five isolates appeared on the plates from each generation were randomly picked for ethanol fermentation and stress tolerance test.

### Isobaric tags for relative and absolute quantitation (iTRAQ) and pathway enrichment analysis

Yeast cells were grown in 25 mL YPD medium for 18 h with an initial OD (A600) of 0.05 to the early stationary phase at which the genes related stress response are greatly induced. The protein content of the yeast cells was extracted by the YeastBuster^TM^ Protein Extraction Reagent (Novagen, Darmstadt, Germany) and quantified by a BCA protein assay kit (Pierce, Rockford, IL, USA). Protein samples were reduced, alkylated, digested with trypsin, and labeled with iTRAQ regents according to the standard iTRAQ protocol (Applied Biosystems, Foster City, CA, USA). Mascot 2.3.02 (Matrix Science, London, United Kingdom) software was used to identity proteins by searching the yeast protein database in Saccharomyces Genome Database (SGD).

### Physiological and biochemical determination

Yeast cells were grown in 25 mL YPD medium under the same condition for iTRAQ experiment. Cells were collected by centrifugation for physiological and biochemical factors extraction. The composition of fatty acids in the strains was analyzed by gas chromatography using a GC FOCUS instrument equipped with a DSQ II MS detector (Thermo, USA) on a DB-5 MS capillary column (J&W Scientific Inc., Folson, CA, USA) as previously described [21]. Ergosterol content was measured using an HPLC system with a reverse-phase column [24]. Prior to HPLC determination, the alkaline hydrolysis of cells (~3×10^8^) in an 3 mL alcohol alkali solution at 95 °C was performed for 3 h followed by extraction in n-heptane. Trehalose was extracted from chilled cells (~1×10^8^) with 3 mL cold 0.5 M trichloroacetic acid (TCA), and the extract was measured for carbohydrate content using the anthrone method [25]. Glutathione (GSH) of yeast cells (~3×10^8^) was extracted from cells with 2 mL 50% ethanol aqueous solution for 2 h, and the extract was measured using a commercial assay kit (Nanjing Jiancheng Biological Products, Nanjing, China). The contents of ergosterol, trehalose, and glutathione are expressed as mg per g dry weight. Catalase (CAT) and superoxide dismutase (SOD) activities were measured as previously described [25].

### Malondialdehyde (MDA) and membrane integrity measurement

Yeast cells (~1×10^8^) were collected and resuspended in 1 mL of 10% TCA. A vortex mixer was used to break up the cells, and the supernatant was used to detect MDA (lipid peroxidation product) through the thiobarbituric acid-reactive species (TBARS) method [10). Cell membrane integrity was observed using a confocal laser scanning microscope (CLSM) after staining with 20 μg/mL fluorescein diacetate (FDA) and 10 μg/mL propidium iodide (PI) for 0.5 h.

### Real-time quantitative PCR (RT-qPCR)

Total RNA from yeast cells samples was extracted using the hot phenol method and then reverse-transcribed into cDNA as previously described [26]. Primer design (Table S1) and RT-qPCR experiments were performed using an ABI Prism 7500 instrument (Applied Biosystems, Foster City, CA, USA). Each sample was tested in triplicate in a 96-well plate (Axygen, Union City, CA, USA) with a final reaction volume of 20 µl using the SYBR^®^ PrimeScript^®^ RT-PCR kit (Takara, Dalian, Shandong, China). The expression activity or the relative copy number of genes was quantified and normalized using the method of Vandesompele et al with *ALG9*, *TAF10*, *UBC6* and *TFC1* as the reference gene [27].

## Results and Discussion

### Comparison of ethanol fermentation performances and stress tolerances of widely used industrial bioethanol *S. cerevisiae* strains

Higher ethanol titer and lower byproduct (such as glycerol and acetic acid) formations are preferred traits for bioethanol *S. cerevisiae* strains. The strains (A1-3, NY1300, NY1308, R12, ZK2, and ZTW1) selected in this study are all industrial *S. cerevisiae* strains used for bioethanol fermentation. Strain ZTW1, which was first isolated from corn mash in our laboratory and used for industrial-scale bioethanol fermentation since 2006, produced more ethanol than the other strains (Table 1). Another advantageous trait of ZTW1 is its lower production of glycerol (0.0337g/g glucose and 0.0354g/g glucose under regular and stressful condition, respectively; Table 1), a main byproduct of ethanol fermentation. The NY1308 and A1 strains showed better tolerance to dehydration and rehydration than other strains, maintaining cell viabilities of 58.4% and 54.2%, respectively (Table 1). These results demonstrate that while the strains are all used in bioethanol production, they show significantly diverse performances in ethanol fermentation and stress tolerance under different conditions.

**Table 1:** The yields of fermentation products and survival rate of widely used industrial *S. cerevisiae* strains.

Strain	Regular condition**^*a*^**				Stressful condition**^*a*^**				Survival rate(%)**^*b*^**
	Ye (g/L)	Yg (g/L)	Ya (g/L)	RS (g/L)	Ye (g/L)	Yg (g/L)	Ya (g/L)	RS (g/L)	
NY1300	105.16±0.94	8.11±0.07	0.62±0.05	1.21±0.25	120.76±0.84	9.72±0.07	0.72±0.05	15.91±0.25	33.2±1.7
NY1308	104.95±0.74	8.36±0.12	0.59±0.09	3.94±0.22	115.95±0.74	9.96±0.12	0.69±0.02	26.74±0.22	54.4±2.2
R12	103.23±0.53	7.65±0.13	0.47±0.06	6.03±0.32	105.83±0.53	8.95±0.13	0.57±0.03	48.23±0.32	14.5±1.5
ZK1	105.58±0.63	7.97±0.06	0.71±0.05	2.09±0.29	113.57±0.91	8.86±0.09	0.71±0.07	31.09±0.29	19.4±1.9
A1	104.97±0.93	8.37±0.07	0.31±0.04	1.54±0.34	119.28±0.63	10.19±0.10	0.49±0.04	19.84±0.27	51.4±2.3
A2	104.17±1.02	8.56±0.09	0.36±0.07	1.72±0.44	118.58±0.53	10.51±0.08	0.45±0.06	21.17±0.36	50.3±2.9
A3	104.57±0.91	8.54±0.08	0.29±0.04	1.32±0.42	118.76±0.79	10.37±0.12	0.42±0.03	21.01±0.34	49.9±2.6
ZTW1	106.08±1.04	7.54±0.11	0.66±0.06	0.52±0.27	122.08±1.04	9.44±0.11	0.66±0.04	13.52±0.14	21.3±1.8

^a^ Fermentation conditions were described in the section of Material and method. Ye, Yg, Ya, and RS indicate the concentration of ethanol, glycerol, acetic acid, and residual glucose, respectively.

^b^ Survival rate means the ratio of viable cells after the treatment of dying and rehydration mentioned in the Material and methods section.

### WGS to improve the ethanol yields and stress tolerance related to ADY production of ZTW1

**Figure 1:**
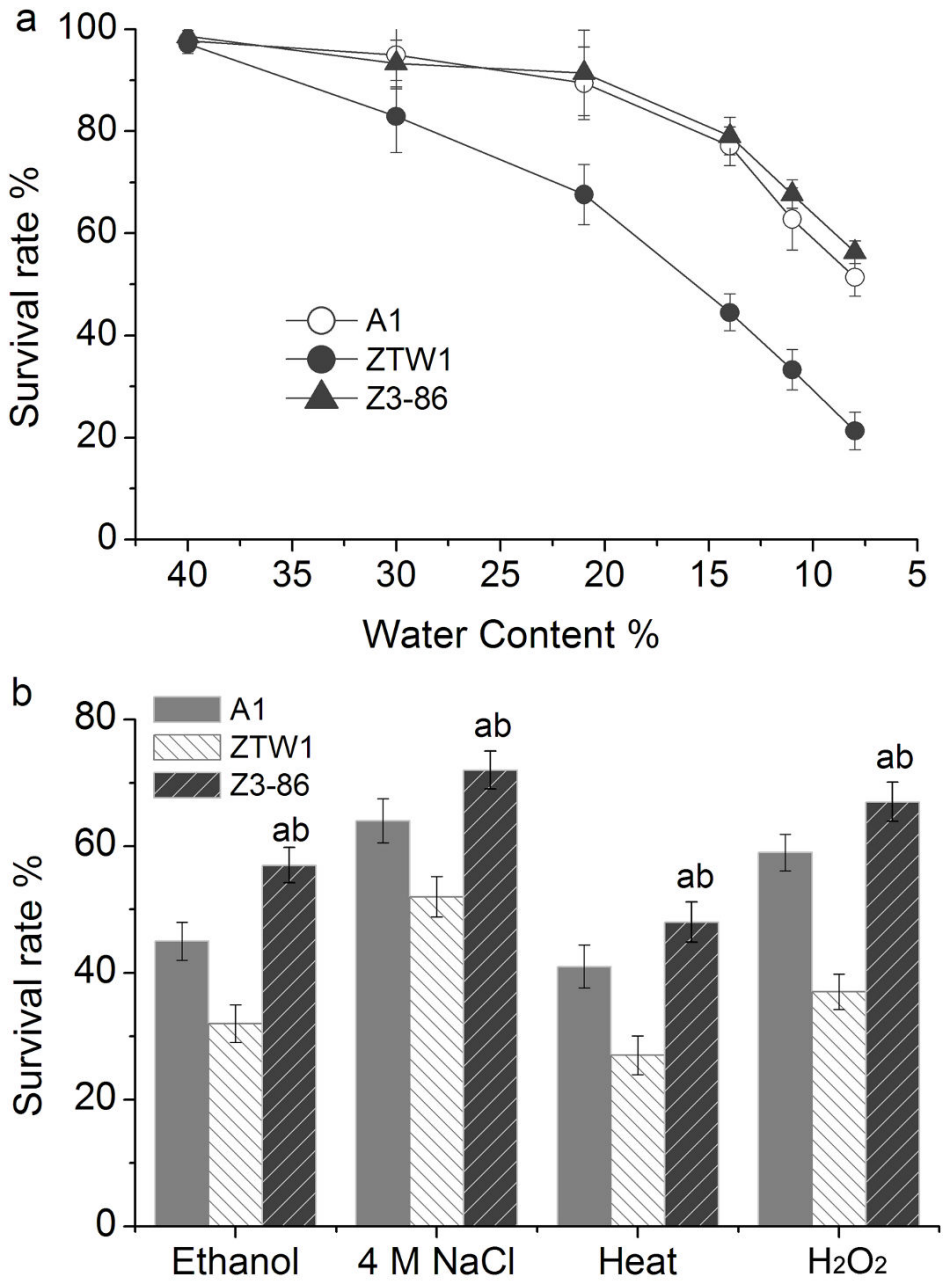
Viabilities of strains A1, ZTW1, and Z3-86 after drying and application of certain stressors. Ratios of viable cells of the three strains after dehydration described in the Material and methods section (a). The dired cells were then diluted serially and the appropriate dilutions were plated onto solid YPD medium to count the colony-forming. The sample without drying was used as the control. Comparison of the tolerance of A1, ZTW1, and Z3-86 to ethanol treatment [20% (v/v) for 2 h], osmotic pressure (4 M NaCl for 2 h), heat (55 °C for 5 min), and H_2_O_2_ (20 mM for 1 h) (b). Yeast cells were pre-cultured in YPD at 30 °C for 18 h with the initial OD(A600) of 0.05. The cells (~3×10^7^) were collected and transferred into 2 mL YPD (for heat tolerance test) or YPD with indicated stressors. The treated cells were diluted and plated onto solid YPD medium for viability calculation. “a” indicates significant difference between Z3-86 and ZTW1 at *P* < 0.01; “b” indicates significant difference between Z3-86 and A1 at the P < 0.05 level, using *t* test. The bars indicated the standard deviation here and other figures.

Although ZTW1 shows higher ethanol titer than commercial ADY strains and other industrial *S. cerevisaie* strains, it was initially consider unqualified for application as an ADY because of its low tolerance to dehydration (Table 1). We sought to address this issue by applying a modified WGS technique to the strain. Unlike in previously reported WGS methods, we first used MBC to treat parent cells and produce genomic variations, after which three rounds of sporulation and hybridization were performed to shuffle the variations in the mutant genomes. Table 2 shows that the ethanol yield and tolerance to desiccation of ZTW1 gradually increased during WGS. The mutant Z3-86, which was obtained from the third round of WGS, produced the highest ethanol among the mutants subjected to stressful conditions (Table 2). The survival ratio of this mutant was 2.15-fold higher than that of ZTW1 after elimination of 92% of its water after drying and slightly higher than that of A1 (Figure 1A; *t* test, *P* < 0.05). 

**Table 2:** The yields of fermentation and survival rate of shuffled strains.

Strains**^*a*^**	Stressful condition**^*b*^**				Survival rate (%)**^*c*^**
	Ye (g/L)	Yg (g/L)	Ya (g/L)	RS (g/L)	
Z1-27	124.73±0.97	10.73±0.12	0.76±0.05	8.12±0.17	51.4±2.6
Z1-89	125.07±1.05	10.82±0.16	0.60±0.06	7.49±0.19	46.6±3.2
Z1-53	124.49±1.21	10.66±0.13	0.65±0.04	8.31±0.26	58.1±3.1
Z2-82	125.82±1.07	10.84±0.11	0.71±0.06	7.21±0.24	57.3±3.4
Z2-76	126.78±1.18	10.56±0.15	0.77±0.07	5.34±0.28	66.7±3.7
Z2-97	124.97±1.02	10.65±0.14	0.69±0.05	8.04±0.39	63.5±2.9
Z3-29	125.98±1.10	10.23±0.17	0.75±0.09	6.99±0.22	59.3±3.4
Z3-86	127.41±1.14	10.91±0.13	0.67±0.08	2.52±0.21	67.1±3.2
Z3-105	126.08±0.99	10.82±0.19	0.62±0.04	5.11±0.17	64.8±2.8

^a^ Three strains with highest ethanol yields from each round of shuffling were selected.

^b^ Ye, Yg, Ya, and RS indicates the concentration of ethanol, glycerol, acetic acid, and glucose, respectively.

^c^ Survival rate means the ratio of viable cells after the treatment of dying and rehydration.

Stress tolerance is crucial for ADY because yeast cells would encounter multiple stressors during ADY production and ethanol fermentation process. Consistent with its improved final ethanol concentration and higher viability after dehydration and rehydration (Table 2), Z3-86 was more resistant to ethanol, osmotic pressure, heat, and H_2_O_2,_ compared with ZTW1. Figure 1b shows that Z3-86 exhibits viability values higher than those of ZTW1 by 78.1%, 38.5%, 77.8%, and 81.1% after treatment with ethanol, osmotic pressure, heat, and H_2_O_2_, respectively (*t* test, *P* < 0.01). Z3-86 also surpassed the viability of A1 with comparative increases in survival rate of 26.7%, 12.5%, 17.1%, and 13.5% after exposure to ethanol, osmotic pressure, heat, and H_2_O_2_, respectively (Figure 1b; t test, *P* < 0.05). These improvements indicate that Z3-86 is more suitable for ADY production than other industrial strains and demonstrates that, in fact, our breeding strategy improves the multiple stress tolerance of yeast. After subculture of Z3-86 for sixty generations as described in the section of Materials and Methods, no significant differences were observed in ethanol titer and tolerances to drying of the progenies (Table S2), indicating Z3-86 can keep a phenotypic stability during serial propagation process. At present, this mutant is being applied in the pilot-scale production of bioethanol ADY in cooperation with TianGuan Technology Co., Ltd., China. Optimization of the ADY production process and the performance of Z3-86 in industrial ethanol fermentation will be discussed in our future reports.

### Proteome analysis to disclose the differently expressed genes between Z3-86 and ZTW1

To investigate how the breeding strategy confers desirable traits to Z3-86, iTRAQ analysis was used to compare the proteomes of this mutant with those of ZTW1. A total of 479 genes showed different expression levels between these two strains (Table S3, *P* < 0.05). Using RT-qPCR, we determined the expression levels of 20 differently expressed genes (DEGs) detected in the iTRAQ experiment. Most of these genes showed consistent expression patterns at the mRNA and protein levels but with changing ratios (Table S1).

Pathway enrichment analysis of DEGs showed that 299 upregulated genes in Z3-86 mainly fall within the pathways of ribosome biosynthesis, proteasome, glycolysis/gluconeogenesis, and secondary metabolite biosynthesis (Table 3; hypergeometric test, *P* < 0.05). A total of 180 downregulated genes were enriched in pathways for oxidative phosphorylation, endocytosis, RNA transport, and vitamin B6 metabolism (Table 3, hypergeometric test, *P* < 0.05). These results suggest that the modified WGS process developed in this study can alter the global transcription pattern of yeast cells, thereby offering a genetic basis for changing the traits of the mutant strain. In the next section, we discuss the possible mechanisms responsible for the different phenotypes of ZTW1 and Z3-86, mainly focusing on DEGs or physiological factors involved in the protein metabolism, membrane composition, trehalose metabolism, and oxidative response of yeast.

**Table 3:** KEGG pathway enrichment of DEGs between ZTW1 and Z3-86.

**Pathways**	**P-value**	**Genes**
**Up-regulated genes**		
Ribosome	1.2E-10	*RPS30A RPS30B RPL21A RPS2 RPS9B RPS13 RPL28 RPL21B RPS9A RPL32 RPL3 RPL23A RPL23B MRPS5 RPS23B RPS23A RPL26B RPL30 RPL18B RPL18A RPL8B RPL38 RPL34B RPL33A RPL16B RPL34A RPS29B RPS11A RPS11B RPL36B RPL16A RPL15A RPL19A RPL19B RPS16B RPS16A RPL2B RPL2A RPS20 RPS26A RPL25 RPL20A RPL20B RPS1B RPS7B*
Proteasome	1.2E-08	*RPN13 SCL1 PRE9 PRE3 RPT6 RPN3 RPN11 PRE5 PUP3 PRE10 PRE7 PRE6 RPN7 RPN5 RPN10 PUP2 PRE4*
Glycolysis/Gluconeogenesis	9.8E-04	*SFA1 PFK2 ADH6 LPD1 FBP1 ADH1 PGI1 TPI1 ADH3 ACS1 ALD2 YMR099C ACS2 TDH1*
Biosynthesis of secondary metabolites	4.9E-03	*ARO8 RKI1 HOM2 HEM13 SFA1 HOM6 ACS2 CAR2 TKL1 YMR099C TRP3 PSA1 TDH1 MDH2 ARG1 LPD1 ADH3 HMG1 FBP1 GAD1 SDH2 PGI1 SOL4 ADH1 ADE2 TPI1 IDH2 ADE16 BAT1 HIS6 AMD1 AGX1 ADH6 HEM2 PFK2 ACS1 GND2 ERG9*
Fructose and mannose metabolism	1.5E-02	*GRE3 FBP1 GCY1 PFK2 TPI1 YPR1 PSA1*
Tyrosine metabolism	2.0E-02	*ARO8 ADH1 ALD2 ADH3 SFA1*
Pentose phosphate pathway	2.2E-02	*RKI1 PFK2 FBP1 PGI1 TKL1 SOL4 GND2*
Methane metabolism	3.8E-02	*SFA1 AGX1 ACS2 PFK2 ACS1 FBP1 DAK1*
Nicotinate and nicotinamide metabolism	4.3E-02	*NPT1 PNC1 QNS1*
**Down-regulated genes**		
Oxidative phosphorylation	2.5E-05	*ATP19 CYT1 COX12 COX6 VMA10 VMA4 ATP14 ACP1 ATP15 QCR6*
Endocytosis	3.0E-03	*DID4 SNF7 DID2 SSA2 GCS1*
RNA transport	2.3E-02	*HCR1 KAP95 SMT3 MEX67 CDC33 TIF4631*
Vitamin B6 metabolism	3.3E-02	*SER1 SNZ3*

### Physiological mechanisms underlying the improved traits of Z3-86 compared with ZTW1

#### Protein metabolism and growth capability

**Figure 2:**
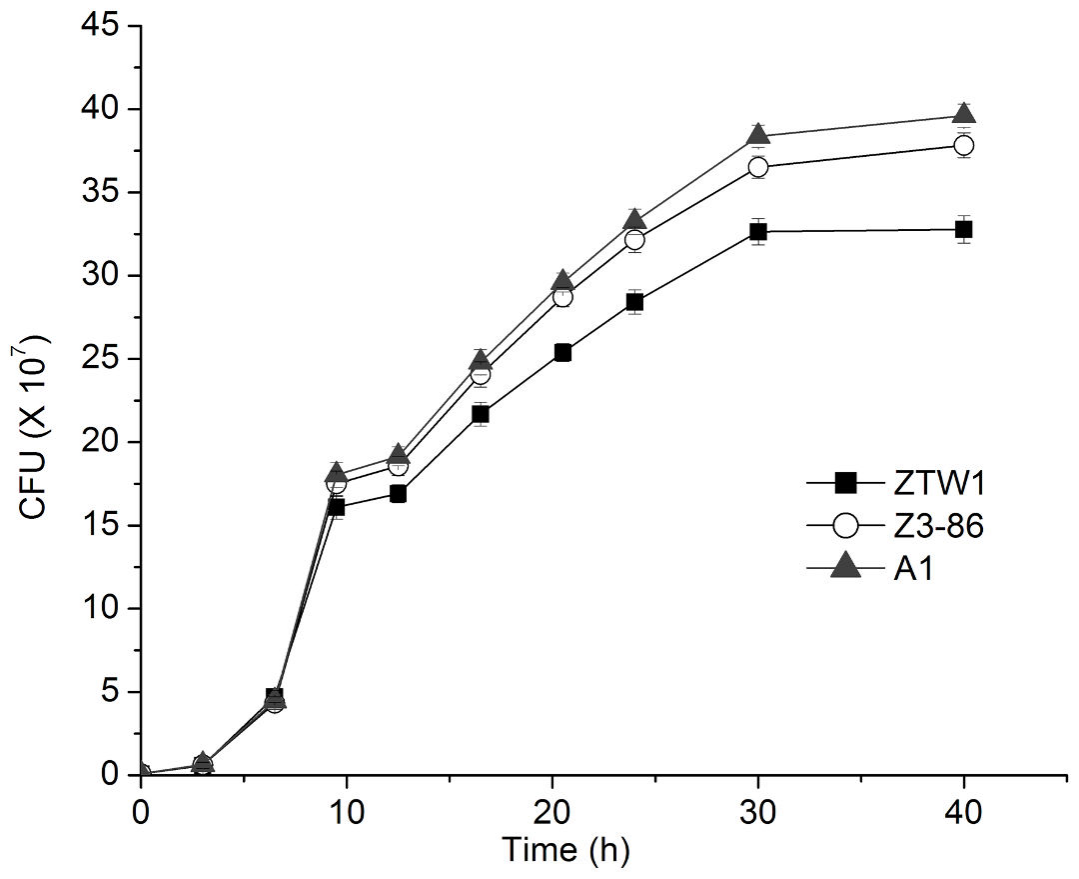
Growth curves of A1, ZTW1, and Z3-86. Cells were cultured in 25 mL of YPD at 30 °C with an initial OD (A600) of 0.05. 20 μL of yeast cells were aspirated at certain time points and the colony forming unites (CFU) were determined. The data was represented as the mean±SD of three independent experiments.

A fairly large number of upregulated genes in Z3-86 that are located in the ribosome and proteasome pathways, which function in protein synthesis and degradation, respectively. Many genes involved in the biosynthesis of secondary metabolites, such as amino acids and nicotinate, were also upregulated in this mutant strain (Table 3). These findings show that the physiological state of Z3-86 tends to promote cell growth and reproduction. In agreement with this speculation, Z3-86 showed higher proliferative capability than ZTW1 (Figure 2). At the stationary phase (40 h), Z3-86 formed 15.4% more cells than ZTW1 and 7.1% less cells than A1 (Figure 2; *t* test, *P* < 0.05), although no significant differences were observed in the dry weights of their biomass (data not shown). These improvements in the growth capability and stress tolerance of Z3-86 are beneficial to ADY production because viable cell count/g yeast solid is an important technical index for finished ADY [7].

##### Plasma membrane integrity and membrane composition

**Figure 3:**
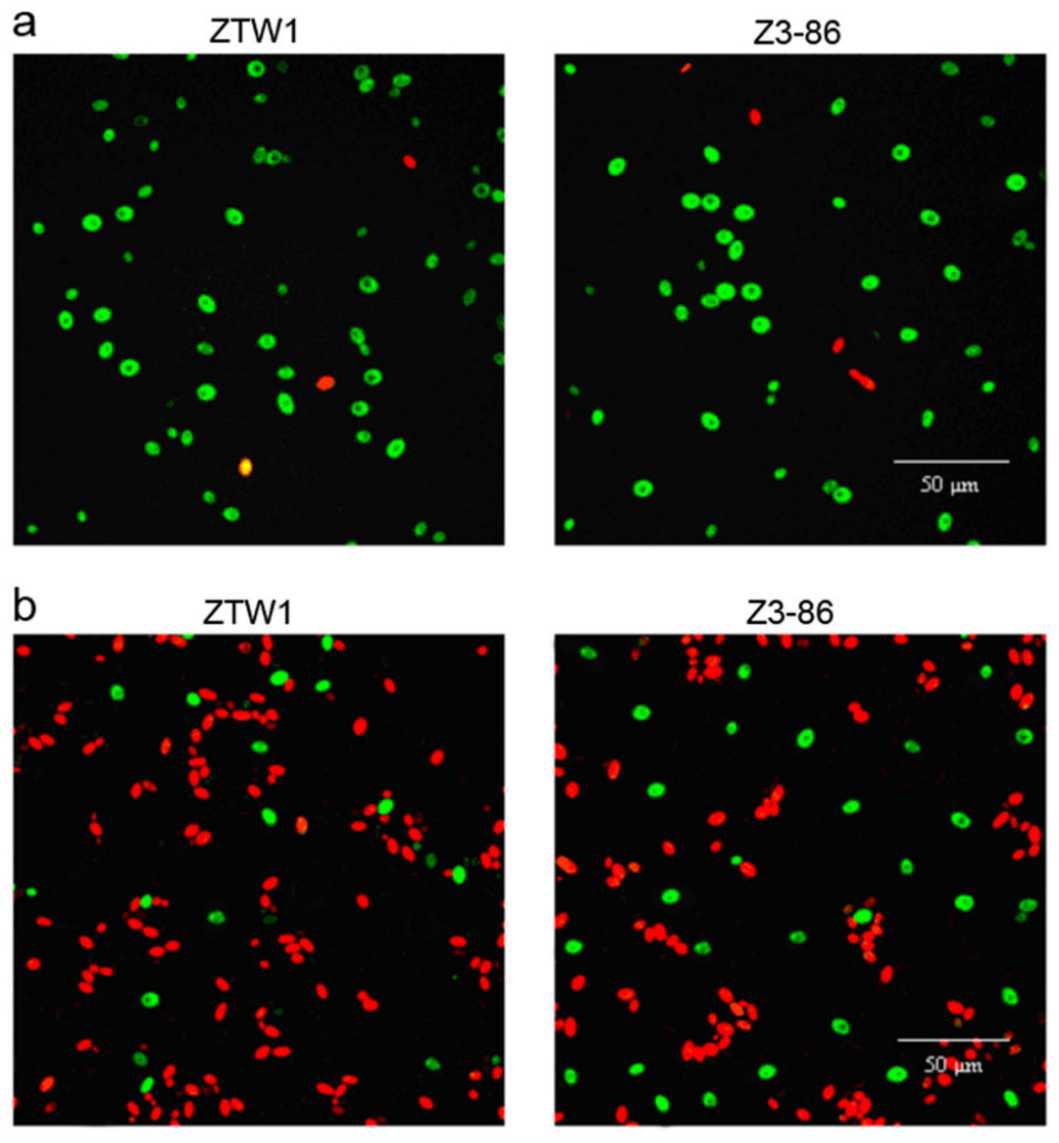
Membrane integrity of ZTW1 and Z3-86. Yeast cells were stained with FDA-PI for 0.5 h and observed using a confocal laser scanning microscope before (a) and after (b) drying. Several view fields were recorded and typical data are shown.

Previous studies report that the plasma membrane is the main target for injury when yeast cells are exposed to environmental stresses [28]. During dehydration and rehydration, water moving across the membrane can largely alter the cell volume and surface-to-volume ratio of yeast cells. As a response, endocytosis and lipid phase transition occur, making the membrane more unstable. Membrane permeabilization is thought to be a critical event leading to cell death during ADY production [29]. To assess the membrane integrity and viability of ZTW1 and Z3-86 after drying, FDA-PI dual fluorescent staining method was used. If the membrane integrity was lost, PI can enter the cell, and make the cell be colored with red by binding the nucleic acid. Viable cells can convert non-fluorescent FDA into a green fluorescent compound, and make themselves be colored green. Figure 3a shows that most of the cells of ZTW1 and Z3-86 were viable and colored with green before drying. Nevertheless, more than half of the cells of these two strains lost their membrane integrity and were stained with PI after drying (Figure 3b). The proportion of ZTW1 and Z3-86 cells stained with PI was 87% and 75%, respectively, indicating the higher ability of the mutant strain to maintain its membrane integrity (Figure 3b).

Membrane composition was previously reported to be closely related to stress-induced membrane permeabilization and cell death [29,30]. Because lipids are more sensitive to osmotic pressure and more compressible than proteins, osmotic pressure induces fatty acyl chains to pack more tightly, reducing membrane fluidity and endangering its integrity [31]. Fatty acid compositions of ZTW1 and Z3-86 were detected under normal and osmotic conditions (1 M NaCl was used as hypertonic dehydrator). Without osmotic pressure, no distinct differences in the four main fatty acid ratios were observed between the two strains (Table 4). By contrast, application of osmotic pressure increased the contents of unsaturated fatty acids (palmitoleic acid, oleic acid, and linoleic acid) in both strains, resulting in 17.6% and 24.6% increases in the unsaturation indices of ZTW1 and Z3-86, respectively (Table 4; *t* test, *P* < 0.05). Z3-86 showed a higher proportion of C_16:1_ and a lower proportion of C_18:1_ compared with ZTW1 (Table 4; *t* test, *P* < 0.05). Higher palmitoleic acid (C_16:1_) contents may be more conducive to the membrane fluidity of Z3-86 under hyperosmolality stress because this fatty acid has a shorter carbon chain than oleic acid.

**Table 4:** Fatty acids composition of ZTW1 and Z3-86.

Strains**^*a*^**	Fatty acid composition (%)**^*b*^**					Unsaturation Index**^*c*^**
	C16:0	C18:0	C16:1	C18:1	C18:2	
ZTW1	25.88±1.75	8.28±1.14	34.65±2.07	31.19±2.34	0.72±0.32	0.68±0.05
Z3-86	27.29±1.82	8.06±1.05	32.92±1.34	31.02±2.16	0.52±0.22	0.65±0.04
ZTW1+NaCl	16.08±1.34	5.88±0.98	36.34±1.29	40.64±1.46	1.34±0.46	0.80±0.03
Z3-86+NaCl	18.05±1.42	6.21±1.12	40.89±1.64	37.72±1.98	1.12±0.37	0.81±0.04

^a^ Yeast cells were grown in YPD for 18 h and transferred to YPD with or without 1 NaCl for 6 h. Fatty acids were determined as described in Material and methods.

^b^ Fatty acid are shown by the number of carbon atoms: number of unsaturated linkages.

^c^ Unsaturation Index (Δ/mol) was calculated as: Δ/mol (1×(% monoene) + 2×(% diene) )/100.

**Figure 4:**
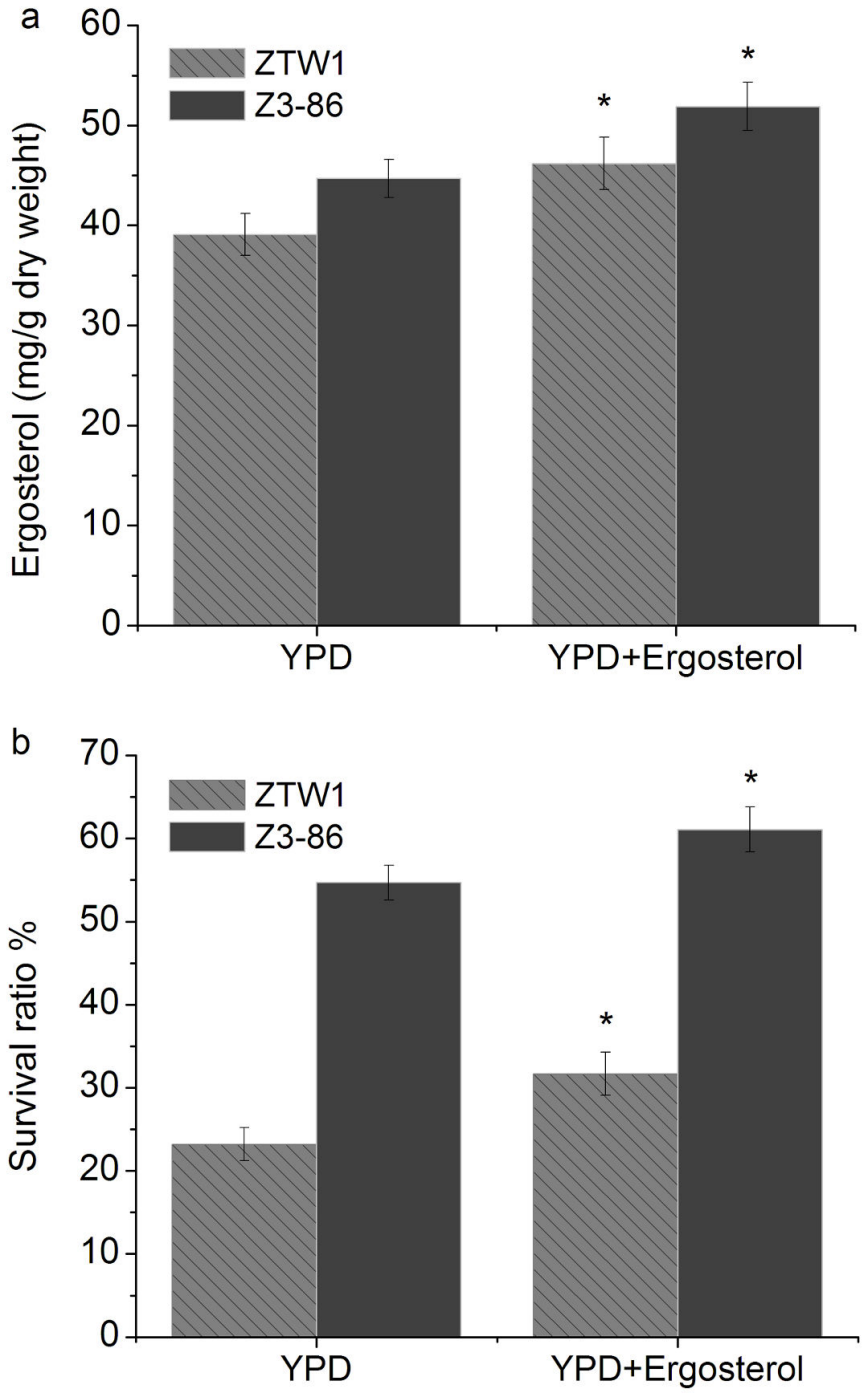
Ergosterol contents and survival ratios of ZTW1 and Z3-86. Ergosterol contents of the strains when grown [initial OD (A600)=0.05] in 25 mL YPD or 25 mL YPD with 20 mg/L ergosterol (a). Ergosterol was first dissolved in hot ethanol and then added to the YPD medium. More ergosterol contents in the plasma membrane improved the survival ratio of yeast cells after drying (b). “*” indicates significant difference between samples at the P < 0.05 level, using *t* test.

Ergosterol has been demonstrated to play a role in membrane stabilization by suppressing the phase transition of phospholipid bilayers [32]. iTRAQ data revealed that two genes involved in ergosterol synthesis, *HMG1* (HMG-CoA reductase annotated in the SGD database; catalyzes the conversion of HMG-CoA to mevalonate, which is a rate-limiting step in sterol biosynthesis) and *ERG9* (encoding farnesyl-diphosphate farnesyl transferase), were upregulated in Z3-86. Compared with ZTW1, the mutant strain accumulated 14.6% more ergosterol (Figure 4a). Additional ergosterol in the culture medium enhanced ergosterol contents in ZTW1 and Z3-86 by 18.4% and 16.1%, respectively (Figure 3a), and the survival ratio of these two strains was accordingly enhanced by 8.5% and 6.4%, respectively (Figure 4b). These results indicate that an altered membrane composition may partially explain the changed traits of the mutant Z3-86.

#### Intracellular trehalose content

**Figure 5:**
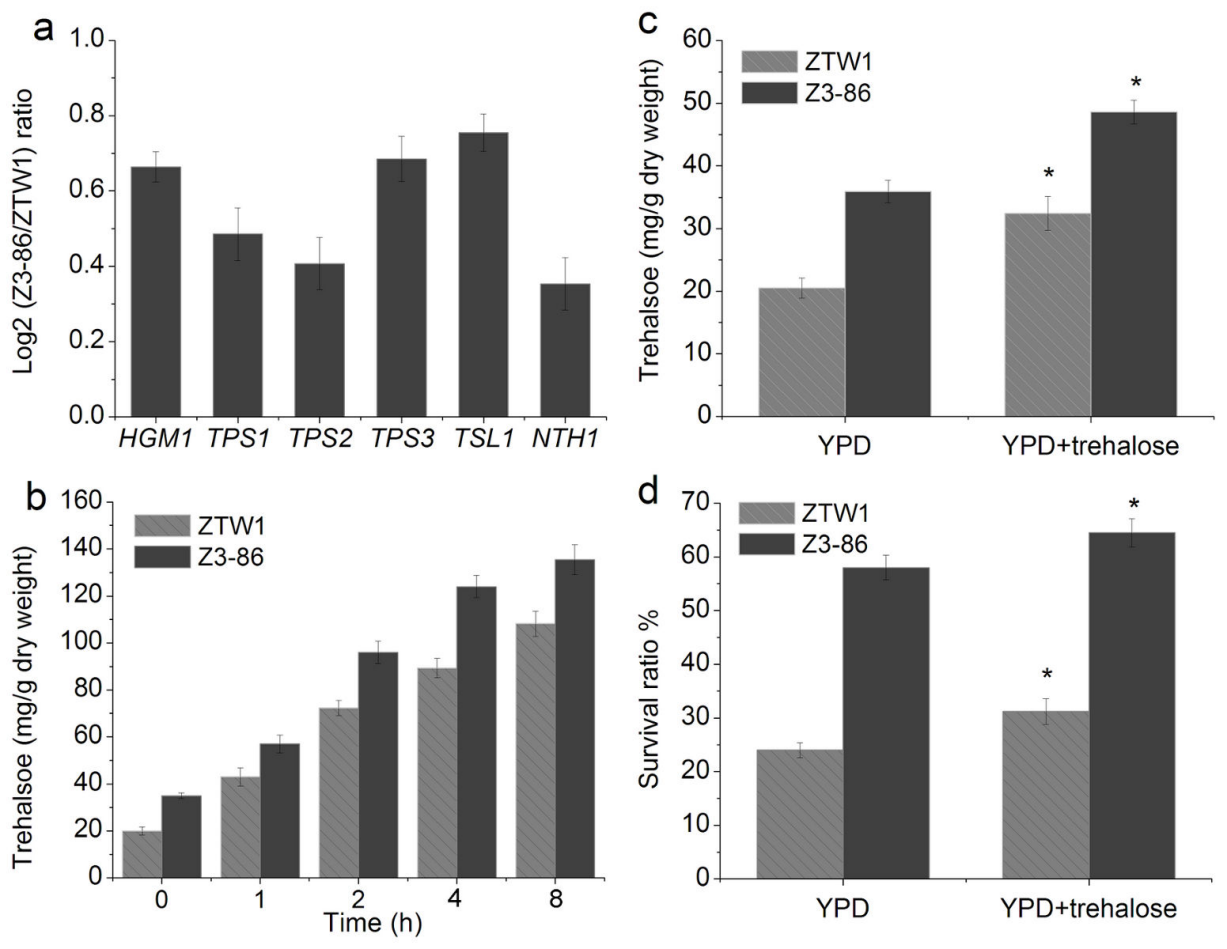
Differences in the trehalose metabolism of ZTW1 and Z3-86. Genes involved in trehalose metabolism were upregulated in Z3-86 (a). Osmotic pressure induced intracellular trehalose accumulation in ZTW1 and Z3-86 (b). Trehalose contents of ZTW1 and Z3-86 when cultured in YPD or YPD with 50 mM trehalose for 18 h (c). Exogenous trehalose enhanced the viability of ZTW1 and Z3-86 (d). “*” indicates significant difference between samples at the P < 0.05 level, using *t* test.

Trehalose is present in a wide variety of organisms and can protect proteins and cellular membrane from inactivation and denaturation caused by many stressful conditions. In addition to *TSL1* and *NTH1* genes, which were detected in the iTRAQ experiments, other four genes (*PGM1*, *TPS1*, *TPS2*, and *TPS3*) involved in trehalose metabolism also showed higher expression levels in Z3-86 than in ZTW1, as determined by RT-qPCR (Figure 5a). Osmotic pressure greatly induced trehalose synthesis in both ZTW1 and Z3-86, but the mutant generally accumulated more trehalose than ZTW1 at different time points (Figure 5b). At the 8 h time point, the trehalose contents in ZTW1 and Z3-86 respectively reached 10.8% and 13.5% of their dry weights (Figure 5b). After being cultured in YPD medium with exogenous trehalose, an increased intracellular trehalose content was observed in both the strains, although Z3-86 synthesized more trehalose than ZTW1 (Figure 5c). The survival ratio of ZTW1 and Z3-86 was correspondly increased by 8.3% and 6.8%, respectively, after air-drying (Figure 5d). These results were consistent with the findings of Gadd et al. that trehalose-enriched yeast cells have stronger tolerance to dehydration and desiccation than cells without enrichment [33]. The protective role of trehalose may be embodied by its substitution of water as well as its binding to the polar head groups of phospholipids and proteins to maintain the biological functions of these molecules under stressful conditions [34]. Thus, increased accumulation of intracellular trehalose may contribute to the higher cell viability of Z3-86 after dehydration and rehydration.

#### Oxidative damage and anti-oxidative factors

**Figure 6:**
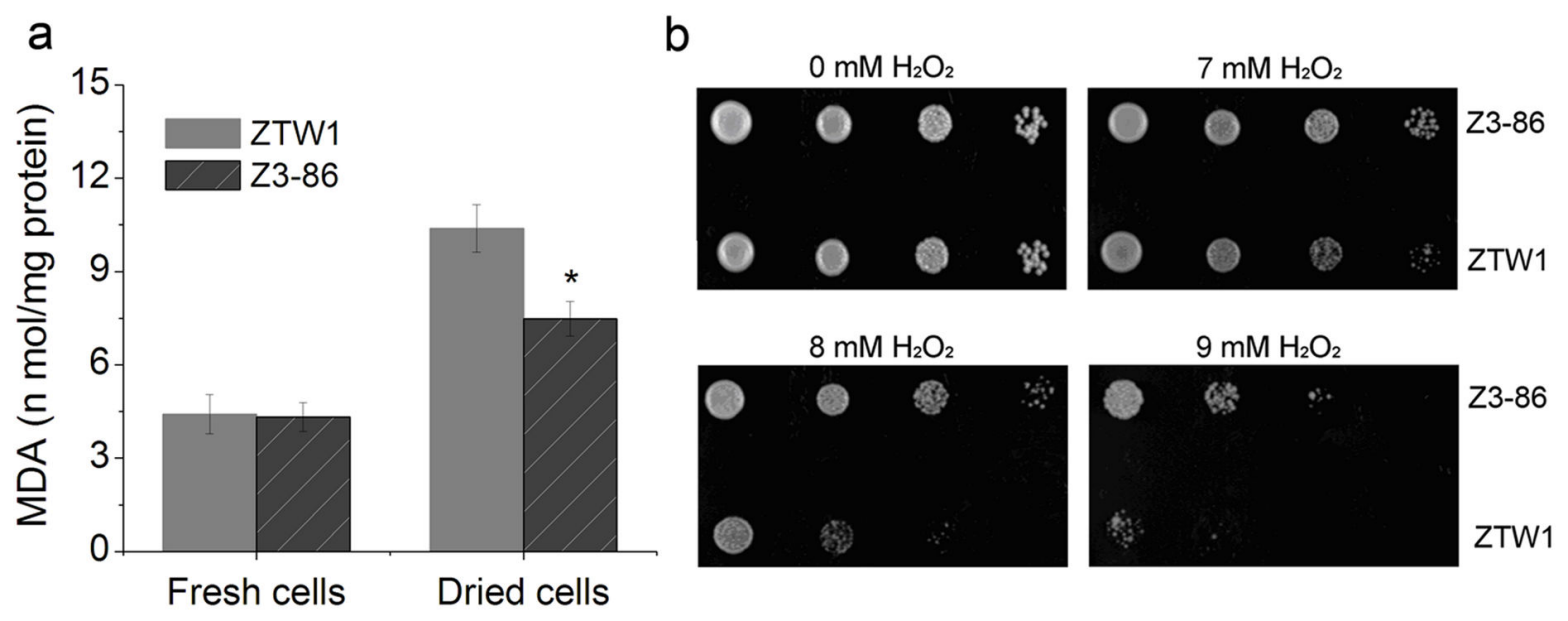
Malondialdehyde (MDA) formation and tolerance to H_2_O_2_ of ZTW1 and Z3-86. MDA content determination of fresh and dried ZTW1 and Z3-86 cells (a). “*” indicates significant difference between samples at the P < 0.05 level, using *t* test. Growth of ZTW1 and Z3-86 on YPD plates containing H_2_O_2_ (b). Cells were pre-cultured in YPD liquid medium for 18 h, and 5 μL of 10-fold serial dilutions were spotted onto plates.

Reactive oxidative oxygen (ROS) caused by the drying process is another main stress for ADY strains [10,35,36]. ROS is injurious to biomolecules such as DNA and protein and can induce lipid peroxidation in yeast cells [37,38]. Under regular conditions, low levels of MDA resulting from lipid peroxidation formed in both ZTW1 and Z3-86. Dehydration led to a significant increase in MDA levels in both strains, although Z3-86 showed 28.0% lower MDA concentrations than ZTW1 (Figure 6a). The mutant also exhibited superior growth compared with Z1 when grown on YPD plates with H_2_O_2_ (Figure 6b). These results corroborate the conclusion that Z3-86 is more tolerant to oxidative stress than its parental strain.

The radical and peroxide scavenging enzymes (such as SOD and CAT), the thioredoxin system, and the glutathione/glutaredoxin system help maintain the reduced environment of yeast cell and defend the cell against oxidative stress caused by multiple stressors [39]. As shown in Table 5, compared with ZTW1, Z3-86 has slightly lower SOD and similar CAT activities; these findings suggest that the mutant strain may not show greater antioxidant potential in terms of antioxidant enzymes. iTRAQ data showed that *TRX2* gene, which is involved in the thioredoxin system, and *GSH2* and *GLR1* genes, which are involved in the glutathione/glutaredoxin system, were significantly upregulated in Z3-86 (Table S3). An additional seven genes upregulated in Z3-86 were found to be involved in the pentose phosphate pathway, which functions in NADPH formation and serves as an electron donor for oxidized glutaredoxins and GSH (Table 2 and Table S3). Consistent with these findings, Z3-86 showed 38.2% and 27.5% more GSH than ZTW1 under both normal and osmotic conditions than ZTW1 (Table 5; *t* test, *P* < 0.05). Although enzymatic antioxidant systems are effective against ROS under conditions of sufficient water, only molecular antioxidants such as GSH and trehalose can alleviate oxidative stress when water is insufficient [40]. These molecular antioxidants immobilize the cytoplasm to a glassy state, thereby preventing chemical reactions, molecular diffusion, and conformational changes in biomolecules and protecting cell membranes under water deficiency [41]. These observations suggest that improved antioxidative abilities related to trehalose, thioredoxin, and glutathione production are important mechanisms in the improved multiple stress tolerance of Z3-86.

**Table 5:** The activities or contents of anti-oxidative factors in ZTW1 and Z3-86.

Strains**^*a*^**	SOD(U/mg prot)	CAT(U/mg prot)	GSH (mg/g)
ZTW1	164±6.14	35±1.42	4.76±0.49
Z3-86	147±4.52	37±1.87	6.58±0.37
ZTW1+NaCl	191±3.96	43±2.12	6.76±0.28
Z3-86+NaCl	182±4.28	45±1.95	8.62±0.35

^a^ Yeast cells were grown in YPD for 18 h and transferred to YPD with or without 1 NaCl for 2 h. SOD, CAT, and GSH were determined as described in Material and methods.

## Conclusion

In the present study, a modified WGS approach was developed to improve the traits of *S. cerevisiae* ZTW1 related to ADY production. The mutant strain Z3-86 was determined to be qualified as a commercial ADY because of its improved multiple stress tolerance. Comparative analysis of Z3-86 and ZTW1 indicated that the increased protein metabolism capability, membrane integrity, trehalose content, and anti-oxidative activities of Z3-86 are important mechanisms underlying its altered phenotypes. This work reports the construction of a novel bioethanol ADY for the first time and enriches the current understanding of how WGS improves the complex traits of microbes.

## Supporting Information

Table S1 **Primers used to determine the expressions of genes through RT-qPCR.** A total of 20 genes were selected to determined their expression levels at mRNA levels using RT-qPCR. These primers were designed using the software Primer Premier 5.0. Most of these genes had consistent expression patterns at the mRNA and protein levels.
(DOC)

Table S2 **Phenotypic comparison of the progenies of Z3-86 after serial passage.** A total of 15 progenies of Z3-86 were selected for ethanol fermentation and survival rate test after drying. There were no significant differences in ethanol titer and tolerances to drying of the progenies.
(DOC)

Table S3 **iTRAQ analysis of ZTW1 and Z3-86.** The differently expressed genes between ZTW1 and Z3-86 were determined by iTRAQ experiment. “*” indicates significant difference between samples at the P < 0.05 level.
(XLS)
