# Supplementary material for: Construction of Novel Saccharomyces cerevisiae Strains for Bioethanol Active Dry Yeast (ADY) Production
Source: PLoS One. 2013 Dec 23;8(12):e85022. doi: 10.1371/journal.pone.0085022 (PMC3871550; doi:10.1371/journal.pone.0085022)
Supplement: Table S1 — Primers used to determine the expressions of genes through RT-qPCR. A total of 20 genes were selected to determined their expression levels at mRNA levels using RT-qPCR. These primers were designed using the software Primer Premier 5.0. Most of these genes had consistent expression patterns at the mRNA and protein levels. (DOC) [file pone.0085022.s001.doc]

**Table S1** Primers used to determine the expressions of genes through RT-qPCR.

| Primer number | Name | Sequence 5'-3' | Size (bp) | Efficiency^a^ | Ratio ^b^ |
| --- | --- | --- | --- | --- | --- |
| 1 | MNP1-F | ACTACAACAGCACCATCACATAA | 290 | 0.98 | 0.78 |
|  | MNP1-R | GAGTCCAACTTCACCGTAAACA |  |  |  |
| 2 | NTH1-F | TACAACACAAAAATCAAGCA | 270 | 1.02 | 0.92 |
|  | NTH1-R | TAAATAACCATAAGAACGGA |  |  |  |
| 3 | TRX1-F | TTACTCAATTCAAAACTGCCA | 145 | 0.95 | 0.69 |
|  | TRX1-R | GCTTGTGGGTATTGTTCAGAG |  |  |  |
| 4 | PRE5-F | ATCATCAAATGTGACGAGCA | 212 | 0.94 | 0.93 |
|  | PRE5-R | AACAAACCAACACCGTAGGG |  |  |  |
| 5 | TKL1-F | GGTCCAACACATCAACCTATT | 279 | 0.96 | 0.82 |
|  | TKL-R | AACACTCAAAGACACTTCGGA |  |  |  |
| 6 | ADE2-F | AATGTGAACAAAGGCTGAACTA | 267 | 0.97 | 0.92 |
|  | ADE2-R | ATCCTATGTGGAGTTCTATGAG |  |  |  |
| 7 | HMG1-F | GGTGATACTACGAGAGCGGTTG | 274 | 0.94 | 0.79 |
|  | HMG1-R | CAGCATTGATTGCCTTACAGCC |  |  |  |
| 8 | PAA1-F | ACCTGGCTACTTTACTTTTA | 222 | 1.03 | 1.21 |
|  | PAA1-R | AGTTGTCGTATTCTTCCTTA |  |  |  |
| 9 | ATP20-F | GCCGAGATTTCAAAACAGA | 213 | 1.02 | 0.62 |
|  | ATP20-R | ACCGACAGAATAAAACCCT |  |  |  |
| 10 | GET4-F | TAAAGGCGAAGCAGGGCGGT | 232 | 0.98 | 1.27 |
|  | GET4-R | CCAATGGTGTTGTGCAAGTAAG |  |  |  |
| 11 | ALD2-F | TTGAGGAGGAGCAAGACACAC | 209 | 0.95 | 0.75 |
|  | ALD2-F | AGCAACAACGCCAAAAGGA |  |  |  |
| 12 | PHO8-F | ACTCACACATTACCAAGCGAAC | 140 | 0.97 | 0.68 |
|  | PHO8-R | GCAACAAACCAATACAGACCAC |  |  |  |
| 13 | ACP1-F | AGCAAATCTCCAGCGATACCCA | 138 | 0.94 | 0.81 |
|  | ACP1-R | CAACACTTCTCAACTCATCAGCCA |  |  |  |
| 14 | SOP4-F | GAAACTGGCGGGCGTTATTA | 107 | 0.99 | 1.31 |
|  | SOP4-R | TTCTTTTTGCCTCCTCTCTGA |  |  |  |
| 15 | SFA1-F | TGGGGGTCTGGATTTTACTT | 299 | 1.06 | 0.53 |
|  | SFA1-R | AGGCTTGATTGATTTCTTTGA |  |  |  |
| 16 | DLD1-F | CAGATACCTACTTCAACACGCA | 298 | 0.98 | 0.84 |
|  | DLD1-R | AAATAGTCATTCAAATCCTCCC |  |  |  |
| 17 | SNZ3-F | GCCGTCAAGCACATCACCAAGA | 178 | 0.94 | 1.19 |
|  | SNZ3-R | TAGCAACTCCACCAGCAGCAAA |  |  |  |
| 18 | TSL1-F | AACAACCTCCAGCCTCTCCCA | 123 | 0.97 | 0.97 |
|  | TSL1-R | GCCCTTCACTTGCTTCATCAT |  |  |  |
| 19 | RPL9A-F | AGCATCAAGTCCAGAATCGTCA | 171 | 1.07 | 0.78 |
|  | RPL9A-R | TGGCACCATCCTTTTCAACAAT |  |  |  |
| 20 | FES1-F | CTAATAAGAAACCACAAAGATA | 272 | 0.95 | 1.09 |
|  | FES1-R | AGGTGAGAGAGAAAAGACAGAA |  |  |  |

a Efficiency (E) was determined using the formula E=10(-1/slope)-1,with the “slope” being the slope of the standard curve that was obtained from 10 fold serial dilution of template.

b Ratio represents the value of (Z3-86/ZTW1)RT-qPCR/(Z3-86ZTW1)iTRAQ of a specified gene, reflecting the consistency of the expression data of RT-qPCR and iTRAQ.
