# Supplementary material for: Construction of Novel Saccharomyces cerevisiae Strains for Bioethanol Active Dry Yeast (ADY) Production
Source: PLoS One. 2013 Dec 23;8(12):e85022. doi: 10.1371/journal.pone.0085022 (PMC3871550; doi:10.1371/journal.pone.0085022)
Supplement: Table S2 — Phenotypic comparison of the progenies of Z3-86 after serial passage. A total of 15 progenies of Z3-86 were selected for ethanol fermentation and survival rate test after drying. There were no significant differences in ethanol titer and tolerances to drying of the progenies. (DOC) [file pone.0085022.s002.doc]

| Strain ^a^ | Stressful condition ^b^ | | | |  | Survival  rate (%) ^c^ |
| --- | --- | --- | --- | --- | --- | --- |
|  | Ye (g/L) | Yg (g/L) | Ya (g/L) | RS (g/L) |  |  |
| Z3-86 | 127.41±1.14 | 10.91±0.13 | 0.67±0.08 | 2.52±0.21 |  | 67.1±3.2 |
| P1 | 126.64±1.07 | 10.52±0.14 | 0.62±0.07 | 3.53±0.24 |  | 66.4±3.1 |
| P2 | 127.34±1.12 | 10.78±0.11 | 0.69±0.08 | 2.34±0.22 |  | 65.9±2.8 |
| P3 | 127.12±1.05 | 10.92±0.09 | 0.64±0.06 | 2.37±0.25 |  | 67.6±3.4 |
| P4 | 126.77±1.13 | 10.67±0.12 | 0.65±0.09 | 2.60±0.27 |  | 68.2±2.2 |
| P5 | 126.93±1.19 | 10.68±0.15 | 0.64±0.08 | 2.72±0.21 |  | 65.8±2.7 |
| P6 | 126.82±1.16 | 10.84±0.19 | 0.67±0.05 | 3.04±0.26 |  | 66.1±3.1 |
| P7 | 127.61±1.22 | 10.87±0.16 | 0.65±0.07 | 2.45±0.22 |  | 66.8±3.0 |
| P8 | 126.59±1.37 | 10.74±0.13 | 0.62±0.05 | 3.04±0.19 |  | 66.4±3.3 |
| P9 | 127.73±1.15 | 10.93±0.12 | 0.65±0.08 | 2.43±0.24 |  | 67.2±3.2 |
| P10 | 127.54±1.22 | 10.68±0.14 | 0.66±0.07 | 2.50±0.32 |  | 66.7±2.7 |
| P11 | 127.12±1.36 | 10.77±0.16 | 0.65±0.04 | 2.57±0.25 |  | 65.2±3.4 |
| P12 | 127.32±1.21 | 10.78±0.13 | 0.67±0.06 | 2.49±0.29 |  | 66.6±3.2 |
| P13 | 127.63±1.18 | 10.95±0.21 | 0.69±0.05 | 2.32±0.27 |  | 67.3±2.7 |
| P14 | 127.19±1.29 | 10.81±0.17 | 0.67±0.08 | 2.52±0.21 |  | 67.5±2.9 |
| P15 | 126.94±1.37 | 10.86±0.18 | 0.64±0.07 | 2.57±0.23 |  | 68.2±3.1 |

**Table S2** Phenotypic comparison of the progenies of Z3-86 after serial passage.

^a^ Strains P1-5 were from the twentieth generation, P6-10 were from the fortieth generation, and P11-15 were from the sixtieth generation.

^b^ Fermentation conditions were described in the section of Material and method. Ye, Yg, Ya, and RS indicate the concentration of ethanol, residual glycerol, acetic acid, and residual glucose, respectively.

^c^ Survival rate means the ratio of viable cells after the treatment of dying and rehydration mentioned in the Material and methods section.
